# Evaluation of Fall and Fracture Risk Among Men With Prostate Cancer Treated With Androgen Receptor Inhibitors: A Systematic Review and Meta-analysis

**DOI:** 10.1001/jamanetworkopen.2020.25826

**Published:** 2020-11-17

**Authors:** Zin W. Myint, Harry D. Momo, Danielle E. Otto, Donglin Yan, Peng Wang, Jill M. Kolesar

**Affiliations:** 1 Division of Medical Oncology, Department of Internal Medicine, University of Kentucky, Lexington; 2 Markey Cancer Center, University of Kentucky, Lexington; 3 University of Kentucky College of Pharmacy, Lexington

## Abstract

**Importance:**

A high incidence of fall and fracture in a subset of patients treated with androgen receptor inhibitors (ARIs) has been reported, although the relative risk (RR) of fall and fracture for patients who receive ARI treatment is unknown.

**Objective:**

To evaluate whether treatment with ARIs is associated with an elevated relative risk for fall and fracture in patients with prostate cancer.

**Data Sources:**

Cochrane, Scopus, and MedlinePlus databases were searched from inception through August 2019.

**Study Selection:**

Randomized clinical trials comparing patients with prostate cancer treated with any ARI or placebo were included.

**Data Extraction and Synthesis:**

Two independent reviewers used a standardized data extraction and quality assessment form. A mixed effects model was used to estimate the effects of ARI on relative risk, with included studies treated as random effects and study groups treated as fixed effects in the pooled analysis. Sample size for each study was used to weight the mixed model. Statistical analysis was performed from August to October 2019.

**Main Outcomes and Measures:**

The primary outcome was RR of fall and fractures for patients receiving ARI treatment.

**Results:**

Eleven studies met this study’s inclusion criteria. The total population was 11 382 men (median [range] age: 72 [43-97] years), with 6536 in the ARI group and 4846 in the control group. Participants in the ARI group could have received enzalutamide, apalutamide, or darolutamide in combination with androgen deprivation therapy or other enzalutamide combinations; patients in the control group could have received placebo, bicalutamide, or abiraterone. The reported incidence of fall was 525 falls (8%) in the ARI group and 221 falls (5%) in the control group. The incidence of fracture was 242 fractures (4%) in the ARI group and 107 fractures (2%) in the control group. Use of an ARI was associated with an increased risk of falls and fractures: all-grade falls (RR, 1.8; 95% CI, 1.42-2.24; *P* < .001); grade 3 or greater fall (RR, 1.6; 95% CI, 1.27-2.08; *P* < .001); all-grade fracture (RR, 1.59; 95% CI, 1.35-1.89; *P* < .001), and likely grade 3 or greater fracture (RR, 1.71; 95% CI, 1.12-2.63; *P* = .01).

**Conclusions and Relevance:**

Use of ARI was associated with an increase in falls and fractures in patients with prostate cancer as assessed by a retrospective systematic review and meta-analysis. Further studies are warranted to identify and understand potential mechanisms and develop strategies to decrease falls and fractures associated with ARI use.

## Key Points

**Question:**

What is the association between androgen receptor inhibitor (ARI) therapy and risk of fall and fracture in men with prostate cancer?

**Findings:**

This systematic review and meta-analysis summarized the results of 11 eligible randomized clinical trials. Use of ARI (enzalutamide, apalutamide, or darolutamide) was associated with an increased risk for all-grade falls and factures, as well as grade 3 or greater falls and fracture.

**Meaning:**

These findings suggest that patients who receive ARI therapy may have a higher risk of fall and fracture; this risk may need to be considered in cancer care.

## Teaser

This systematic review and meta-analysis examines 11 randomized clinical trials to see whether therapy with androgen receptor inhibitors (ARIs), such as enzalutamide, apalutamide, or darolutamide, is associated with an increased risk of falls and fractures in men with prostate cancer.

## Introduction

Fall is one of the top 10 leading causes of death and a common cause of morbidity in the older population.^1^ Risk of fall increases with age. More than one-third of people aged 65 years or older are reported to fall in their community every year, and the risk increases 2-fold at the age of 80.^2^ In 2012, a prospective study^3^ reported that the risk of fall is double in patients with advanced cancer regardless of age. Some underlying predisposing factors for fall risk include history of falls in the last 3 months, severity of depression, use of benzodiazepine, cancer-related pain, and cancer treatment.^3^ Falls can be associated with catastrophic physical injury resulting in bone fractures, head trauma, negative impact on quality of life, and stress for caregivers.

Several antineoplastic therapies are associated with risk for falling. Advanced muscle wasting, called sarcopenia, has been reported to be associated with cancer treatment adverse events.^4^ Several prospective studies have shown that sarcopenia is associated with higher risk for fall, subsequent fractures, and physical disability in patients with cancer.^4^ For example, a prospective, randomized, placebo-controlled study reported that sorafenib, a multikinase inhibitor, was associated with progressive skeletal muscle loss of 4.9% in muscle and fat assessed by computed tomography scans at 6 months and 8% at 1 year.^5^ Other antineoplastic therapies that are associated with sarcopenia include bevacizumab,^6^ fluorouracil-based chemotherapy,^7^ and capecitabine.^8^ Another prospective study^9^ evaluated chemotherapy-induced peripheral neuropathy and the risk of fall in patients receiving taxanes (docetaxel or paclitaxel) and platinum-based chemotherapy (cisplatin or oxaliplatin) in any type of solid cancers, and the results suggested a higher risk for falls assocciated with taxanes than with platinum-based chemotherapy, but the results were not statistically significant (odds ratio = 10.14; *P* = .07).

Similarly, androgen receptor inhibitors (ARIs) are reported to be associated with a higher incidence of falls and fractures in a subset of patients, although a potential mechanism is unclear. We considered and defined ARIs to include enzalutamide, apalutamide, or darolutmide alone or in combinations. We did not consider androgen deprivation therapy (ADT), bicalutamide, or abiraterone as ARIs. This systematic review evaluates the relative risk of fall and fracture in patients with prostate cancer who receive ARIs as defined.

## Methods

We conducted this systematic review and meta-analysis in accordance with the Preferred Reporting Items for Systematic Reviews and Meta-analyses (PRISMA) reporting guideline.^10^ Deidentified variables were collected and no participants were contacted; thus, this study is considered exempt from institutional review board approval and the requirement for informed consent in accordance with 45 CFR §46.

### Search Strategy

We conducted a comprehensive literature search using Cochrane, Scopus, and MedlinePlus databases from inception through August 2019 and evaluated the relevant published studies using the most appropriate free-text term, including *androgen receptor blockers/inhibitors and prostate cancer*, *enzalutamide or darolutamide or apalutamide and prostate cancer*, *androgen receptor blockers/inhibitors and fracture*, *enzalutamide or darolutamide or apalutamide and fracture*, *androgen receptor blockers/inhibitors and fall*, *enzalutamide or darolutamide or apalutamide and fall*, *androgen receptor blockers/inhibitors and clinical trials*, *enzalutamide or darolutamide or apalutamide and clinical trials*, *androgen receptor blockers/inhibitors and phase 2 or phase 3*, and *enzalutamide or darolutamide or apalutamide and phase 2 or phase 3*. A librarian was consulted to ensure the comprehensiveness of the literature search. We carefully selected all published phase 2, phase 3, and phase 4 randomized clinical trials that included reported fall and fractures as adverse events. These data were then extracted for further analysis.

### Study Selection

Two authors (Z.W.M. and H.D.M.) independently screened the relevant studies that were published in a systematic review and a meta-analysis retrieved from the search results of the Cochrane, Scopus, and MedlinePlus databases. We selected the most appropriate studies on the basis of our inclusion criteria and independently collected the required data for the individual studies. Discrepancies and disagreements were resolved by discussion with other reviewers (P.W. and J.M.K.) and were finally resolved through consensus of all reviewers.

### Inclusion and Exclusion Criteria

We included all published prospective phase 2, phase 3, and phase 4 randomized clinical trials that used ARIs to treat patients with prostate cancer. Reported falls and fractures as adverse events were extracted for analysis. Retrospective, phase 1, nonrandomized phase 2, and studies with control groups that used 1 of the ARIs were excluded.

### Data Extraction

Two authors (Z.W.M. and H.D.M.) collected the required data from the consensus selected studies. Extracted data from individual studies included trial name, inclusion and exclusion criteria of each included trial, study phase, treatment groups, comparison groups, participant age and race, geographic location, median duration of treatment, total number of participants, reported all-grade fall and fracture adverse events, and reported grade 3 or higher fall and fracture adverse events. We resolved disagreements by consensus of all reviewers. All-grade adverse events are defined as any grade (from grade 1 to grade 4).

### Assessment of Study Quality and Bias Risk

Risk of study quality was assessed using the Joanna Briggs Institute Critical Appraisal Checklist for Randomized Controlled Trials.^11^ The ENZAMET trial^12^ was the only open-label study that lacked blinding between investigators and participants. The remaining trials were multicenter, double-blind, randomized phase 2 and phase 3 trials with well-balanced baseline demographic and clinical characteristics of participation cohorts in all studies. Publication bias was not identified in the studies.

### Statistical Analysis

A mixed-effects model was used to estimate effects of ARI on the relative risk of all-grade fall, grade 3 or greater fall, all-grade fracture, and grade 3 or greater fracture, with the included studies treated as random effects and study groups treated as fixed effects in the pooled analysis. End points were assumed to have binomial distribution and a logistic link function was used in fitting the mixed effects model. The included studies were treated as random effects because heterogeneity is expected among studies. Sample size for each study was used as a weighted mixed-effects model. Pooled relative risks were estimated with 95% CIs. We also calculated the raw relative risks and the corresponding 95% CIs for each individual study. Pooled and raw relative risks are presented. All statistical tests were 2-sided with *P* ≤ .05 to identify statistical significance. Statistical analysis was performed with SAS software version 9.4 (SAS Institute) from August to October 2019.

## Results

**Figure 1.**
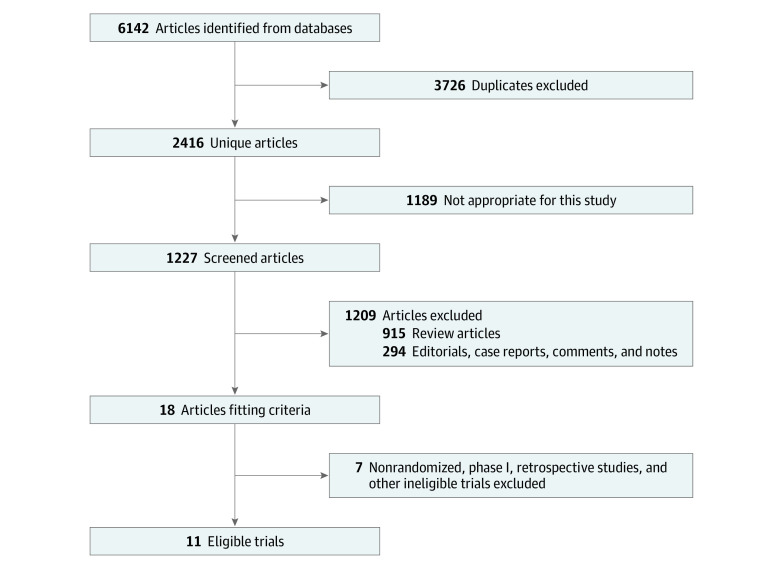
Diagram of Study Selection

A total of 6142 articles were identified in the initial database search. Of these, 3726 were excluded because of duplication. We screened 1227 articles of which 915 were review articles and 294 were case reports. Of the remaining 18 articles, 7 were excluded because of nonrandomized, phase I, or retrospective nature. Eleven studies met our inclusion criteria (Figure 1).

### Basic Characteristics of Included Studies

The total population of the 11 included studies was 11 382 men with a median (range) age of 72 (43-97) years (6536 men were in the ARI group and 4846 men were in the control group). Participants in the ARI group received enzalutamide, apalutamide, or darolutamide in combination with ADT or other enzalutamide combinations. Participants in the control group received placebo, ADT, bicalutamide, abiraterone, or a combination that did not include an ARI, as defined. The breakdown of population by ARI is as follows: 7614 patients were in enzalutamide studies (4250 treatment vs 3364 control), 2259 were in apalutamide studies (1331 treatment vs 928 control), and 1509 were in darolutamide studies (955 treatment vs 554 control).

Disease states included nonmetastatic castration-resistant prostate cancer (nmCRPC), metastatic hormone-sensitive prostate cancer (mHSPC), and metastatic castration-resistant prostate cancer (mCRPC). A total of 8 studies used enzalutamide, 5 of which were phase 3, 2 were phase 2, and 1 was phase 4. Two studies used apalutamide and 1 used darolutamide. The median (range) duration of treatment in the ARI group was 15 (5.4-20.5) months vs 8 (5.4-18.3) months in the control group (Table 1).

**Table 1.** Baseline Characteristics of Included Studies

Study	Phase	Comparison ARI vs control	Patients, No.	Patients, ARI vs control, No.	Cancer status	Age, ARI vs control, median (range), y	Duration of treatment, ARI vs control, median, mo
ARCHES^13^	3 (RCT)	ADT + Enz vs placebo + ADT	1150	574 vs 576	mHSPC	70 (46-92) vs NM	12.8 vs 11.6
STRIVE^14^	2 (RDB)	Enz vs Bical	396	198 vs 198	CRPC	72 (46-92) vs 74 (50-91)	14.7 vs 8.4
PREVAIL^15^	3 (RDB)	Enz vs placebo	1717	872 vs 845	CRPC	72 (43-93) vs 71 (43-94)	18.2 vs 5.4
PROSPER^16^	3 (RDB)	Enz + ADT vs placebo + ADT	1401	933 vs 468	nMCRPC	74 (50-95) vs 73 (53-92)	18.4 vs 11.1
TERRAIN^17^	2 (RCT)	Enz vs Bical	375	184 vs 191	CRPC	67 (50-74) vs 66 (48-74)	12.5 vs 6.0
PLATO^18^	4 (RDB)	Enz + Abi or Abi	251	126 vs 125	CRPC	72 (67-77) vs 71(65-77)	5.6 vs NM
AFFIRM^19^	3 (RDB)	Enz + ADT vs placebo + ADT	1199	800 vs 399	CRPC	NM vs NM	NM vs NM
ENZAMET^12^	3 (RCT)	Enz + ADT vs placebo + ADT	1125	563 vs 562	mHSPC	69 (63-74.5) vs 69 (64-74)	56.2%^a^
TITAN^20^	3(RDB)	Apa + ADT vs placebo + ADT	1052	525 vs 527	mHSPC	69 (45-94) vs 68 (43-90)	20.5 vs 18.3
SPARTAN^21^	3 (RDB)	Apa + ADT vs placebo + ADT	1207	806 vs 401	nMCRPC	74 (48-94) vs 74 (52-97)	60.9%^b^
ARAMIS^22^	3 (RDB)	Dar + ADT vs placebo + ADT	1509	955 vs 554	nMCRPC	74 (48-95) vs 74 (50-92)	14.8 vs 11

Abbreviations: Abi, abiraterone; ADT, androgen deprivation therapy; Apa, apalutamide; ARI, androgen receptor inhibitor; Bical, bicalutamide; CRPC, castration-resistant prostate cancer; Dar, darolutamide; Enz, enzalutamide; mHSPC, metastatic hormone-sensitive prostate cancer; NM, not mentioned; nMCRPC, nonmetastatic castration-resistant prostate cancer; RCT, randomized clinical trial; RDB, randomized double-blind.

^a^ Still receiving treatment at 36 months vs 59.6% still receiving treatment at 36 months.

^b^ Still receiving treatment at median follow-up 20.3 months vs 29.9% still receiving therapy at the median follow-up 20.3 months.

### Outcomes of Fall and Fracture

In the ARI group, the reported incidence of all-grade falls was 525 (8%) and that of grade 3 or greater falls was 62 (1%). In the control group, the reported incidence of all-grade falls was 221 (5%) and that of grade 3 or greater falls was 28 (0.6%). The reported incidence of all-grade fractures in the ARI group was 242 (4%) and that grade 3 or greater fractures was 60 (1%). The reported incidence of all-grade fractures in the control group was 107 (2%) and that of grade 3 or greater fractures was 23 (0.5%). There was no age difference between ARIs and control groups (Table 2 and Table 3).

**Table 2.** Outcomes of Reported Fall and Fractures Adverse Events in Individual Study

Study	Comparison ARI vs control	Patients, No. (%)							
		Fall adverse event				Fracture adverse event			
		ARI group		Control group		ARI group		Control group	
		All grades	Grade ≥3	All grades	Grade ≥3	All grades	Grade ≥3	All grades	Grade ≥3
ARCHES^13^	ADT + Enz vs placebo + ADT	21 (3.7)	2 (0.3)	15 (2.6)	1 (0.2)	37 (6.5)	6 (1)	24 (4.2)	6 (1)
STRIVE^14^	Enz vs Bical	27 (14)	3 (2)	16 (8)	3 (2)	0	0	0	0
PREVAIL^15^	Enz vs placebo	101 (12)	12 (1)	45 (5.3)	6 (0.7)	0	0	0	0
PROSPER^16^	Enz + ADT vs plac + ADT	106 (11)	12 (1)	19 (4)	3 (1)	0	0	0	0
TERRAIN^17^	Enz vs Bical	12 (7)	1 (1)	7 (3)	2 (1)	0	0	0	0
PLATO^18^	Enz + Abi or Abi	0	0	0	0	0	0	0	0
AFFIRM^19^	Enz + ADT vs placebo + ADT	0	0	0	0	0	0	0	0
ENZAMET^12^	Enz + ADT vs placebo + ADT	54 (10)	6 (2)	20 (4)	2 (<1)	38 (7)	16 (3)	13 (2)	5 (1)
TITAN^20^	Apa + ADT vs placebo + ADT	39 (7.4)	4 (0.8)	37 (7)	4 (0.8)	33 (6.3)	7 (1.3)	24 (4.6)	4 (0.8)
SPARTAN^21^	Apa + ADT vs placebo + ADT	125 (15.6)	14 (2.7)	36 (9.0)	3 (0.8)	94 (11.7)	22 (2.7)	26 (6.5)	3 (0.8)
ARAMIS^22^	Dar + ADT vs placebo + ADT	40 (4.2)	8 (0.8)	26 (4.7)	4 (0.7)	40 (4.2)	9 (0.9)	20 (3.6)	5 (0.9)

Abbreviations: Abi, abiraterone; ADT, Androgen Deprivation Therapy; Bical, bicalutamide; Dar, darolutamide; Enz, enzalutamide.

**Table 3.** Pooled Analysis of ARI Use With Fall and Fracture Risk

Adverse event	ARI groups		Control groups		Pool estimate		
	Patients in studies, No.	Patients with adverse events, No.	Patients in studies, No.	Patients with adverse events, No.	Studies, No.	RR (95% CI)	*P* value
Fall							
All grades	6536	525	4846	221	11	1.8 (1.42-2.24)	<.001
Grade ≥3	6536	62	4846	28	11	1.6 (1.27-2.08)	<.001
Fracture							
All grades	6536	242	4846	107	11	1.59 (1.35-1.89)	<.001
Grade ≥3	6536	60	4846	23	11	1.71 (1.12-2.63)	.01

Abbreviations: ARI, androgen receptor inhibitor; RR, relative risk.

When looking at the reported incidence of all-grade falls associated with individual ARI drug, apalutamide had the highest rate at 12% (95% CI, 10.60%-14.21%), followed by enzalutamide at 8% (95% CI, 6.78%-8.39%), followed by darolutamide at 4.2% (95% CI, 3.01%-5.66%) (eTable 1 in the Supplement). Similarly, apalutamide was associated with the highest all-grade fracture rate at 10% (95% CI, 8.0%-11.3%) followed by enzalutamide at 1.8% (95% CI, 1.4%-2.2%) and darolutamide at 4.2%; (95% CI, 3.0%-5.7%) when comparing with each individual ARI drug.

**Figure 2.**
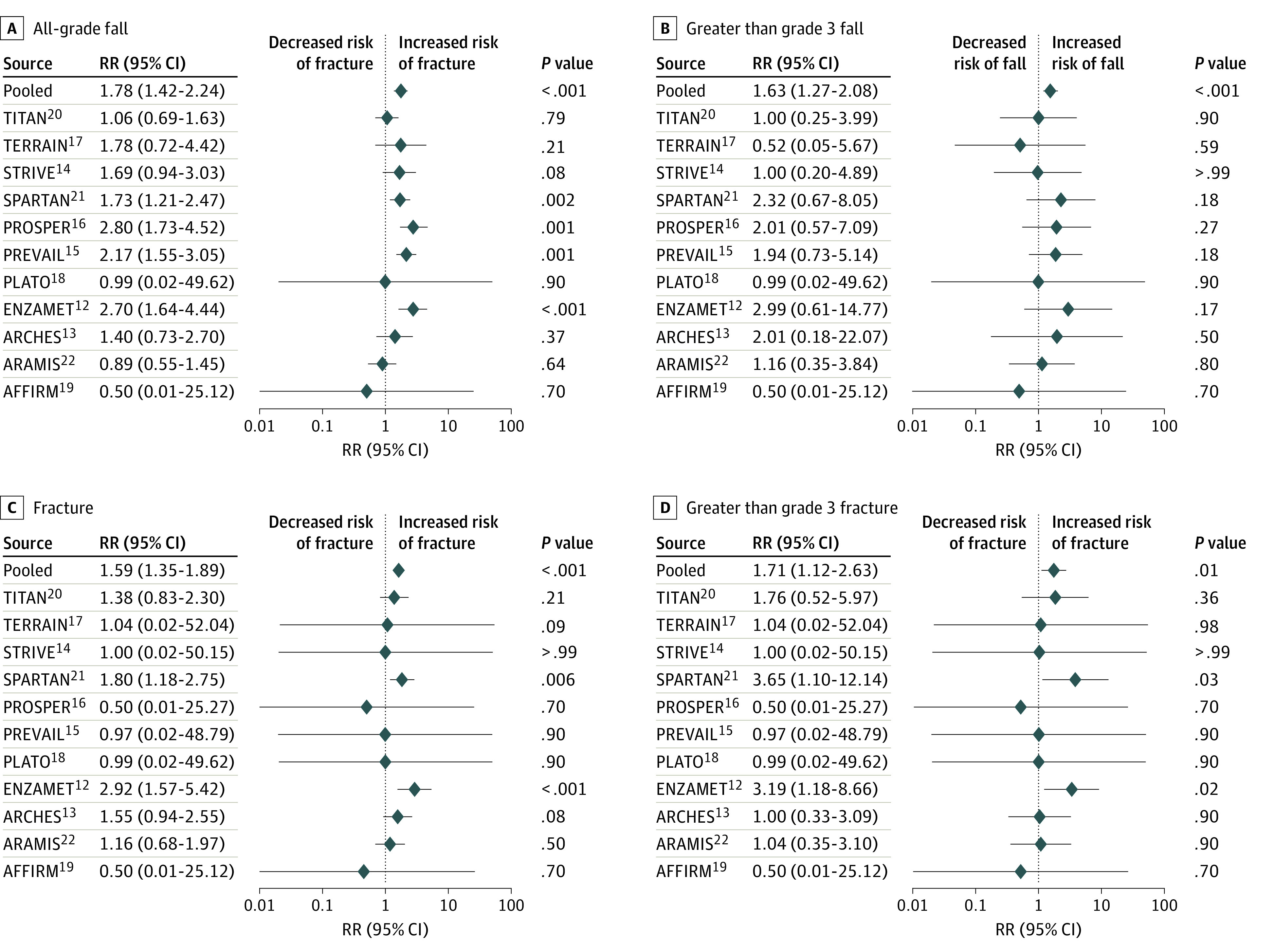
Risk of Fracture or Fall Among Included Studies Graphs show relative risks (RRs) and 95% CIs for all-grade falls (A), greater than grade 3 falls (B), all-grade fractures (C), and greater than grade 3 fractures (D).

In the pooled analysis, use of ARI was associated with an increased risk of all-grade falls (RR, 1.8; 95% CI, 1.42-2.24; *P* < .001), grade 3 or greater fall (RR, 1.6; 95% CI, 1.27-2.08; *P* < .001), all-grade fracture (RR, 1.59; 95% CI, 1.35-1.89; *P* < .001), and likely grade 3 or greater fracture (RR, 1.71; 95% CI, 1.12-2.63; *P* = .01) (Table 3) (Figure 2).

We also investigated whether such an association varied by clinical heterogeneity, such as geographical location, age, race, comorbidities, and inclusion or exclusion differences between studies. A plurality of participants were aged 65 to 74 years (40%-50%); however, we found more participants who were older in the ARMIS (aged 75-84 years, 62%; aged ≥85 years, 14%), PREVAIL (aged 75-84 years, 31%), TERRAIN (aged >75 years, 29%), STRIVE (aged >75 years, 38%), and PLATO (aged >75 years, 40%) studies (eTable 2 in the Supplement). The majority of study participants were from North America and European countries (ARCHES, PREVAIL, TERRAIN, PLATO, and SPARTAN) and Australia and Canada (ENZAMET); however, this information was not provided in some original studies (AFFIRM, TITAN, ARMIS, and others) (eTable 2 in the Supplement).

Patients with comorbidities, such as myocardial infarction, congestive heart failure, ventricular arrythmia, unstable angina, heart block, bradycardia, uncontrolled hypertension, and seizure disorders, were excluded from all studies. Patients in PROSPER, AFFIRM, PLATO, SPARTAN, and ARMIS studies were stratified according to usage of baseline bone-health agents (reported as 10%, 43%, 22%, 10%, and 7%, respectively) (eTable 2 in the Supplement).

## Discussion

This systematic review and meta-analysis of 11 randomized clinical trials further supports the association between use of ARIs and risk of fall and fracture.^12,13,14,15,16,17,18,19,20,21,22^ The use of ARIs is associated with 1.8 times higher risk of fall and 1.6 times higher risk of fracture. ARIs are novel hormonal agents with substantial overall survival improvement in patients with nmCRPC, mHSPC, and mCRPC. All 3 ARIs are nonsteroidal androgen receptor antagonists; enzalutamide and apalutamide have similar molecular structure with high affinity for the ligand-binding domain of androgen receptors.^23^ However, darolutamide has a unique molecular structure; its active metabolite inhibits androgen receptor translocation and testosterone-induced downstream effects of DNA activation, prostate cancer cell growth, and survival.^24^

In the enzalutamide PREVAIL study, the higher incidence of fall (19.2% vs 7.2%) and fracture (15.8% vs 9.9%) was seen in patients aged 75 years and older compared with patients aged 74 years and younger.^25^ To date, it is unclear why the ARI drug class is associated with higher risk of fall. One of the possible explanations is its ability to cross the blood-brain barrier (BBB). Both enzalutamide and apalutamide have this ability and thus can be considered for use in brain metastasis.^19^ In the ARMIS trial, when comparing darolutamide with placebo, the reported incidence of fall and fracture with darolutamide was even lower than placebo.^22^ Tissue distribution of BBB penetration by using ^14^C-labeled whole-body autoradiography and comparing darolutamide vs enzalutamide in an animal model demonstrated that darolutamide has a 10-fold lower BBB penetration than enzalutamide with fewer central nervous system (CNS) adverse effects, including falls.^26^ Comparing the brain-plasma concentration ratio between apalutamide (ARN-509) and enzalutamide (formerly MDV3100) in LNCaP xenograft mice after 28-day treatment demonstrated that the brain-plasma concentration ratio of apalutamide was 4-fold lower than enzalutamide, suggesting a lower threshold for clinical seizures and CNS toxicities.^27^ Therefore, theoretically, enzalutamide has the highest CNS toxicity rates followed by apalutamide followed by darolutamide.

Another possible explanation is that the sarcopenia associated with ARIs has a higher risk for fall. One phase 2 study showed that enzalutamide monotherapy was associated with a 4.2% decrease in mean body mass index.^28^ There was a 22% increased risk of visceral abdominal fat after 12 months of treatment with ADT.^29^ Thus, dual hormone blockage (ADT combined with ARIs) may be associated with higher risk of muscle loss and sarcopenia.

Other possibilities are the use of concomitant medications (such as benzodiazepines or opioid medications), fatigue from disease and/or as an adverse effect of ARIs, underlying predisposing conditions (such as cognitive impairment, depression, or multiple medical comorbidities), poor performance status, and history of falls.

One Canadian study^30^ surveyed older patients (≥65 years) who were receiving active cancer treatment and their oncologists to better understand how falls are associated with cancer care interruptions. One of the interesting findings was that 7% of reported fall cases were attributable to cancer treatment interruption, including chemotherapy and/or androgen deprivation therapy, but not reported with androgen receptor blocker agent interruption.^30^

Data on use of bone-health agents were not available for all studies, so our study could not make a strong conclusion on whether using bone-health agents would reduce the rate of fracture. Denosumab, an anti-RANKL monoclonal antibody, was shown to delay the time to first bone metastasis in nmCRPC; however, there was no benefit in progression-free survival or overall survival and it did not prevent pathological fracture.^31^ Thus, denosumab was not approved for use by the US Food and Drug Adminstration in this context. However, denosumab is approved for use in patients with mCRPC who have bone metastases,^32^ and it is also approved to use for ADT-associated bone loss in patients with prostate cancer to improve bone mass.^33^

There are multiple validated fall-risk assessment tools in noncancer populations. For example, the Hendrich II Fall Risk Model is a validated tool to use in acute care, ambulatory, and inpatient settings to determine the risk of fall and for secondary prevention of falls.^34,35^ The 12-item Falls Risk Questionnaire is a fall-risk screening tool in noncancer populations and it has also been useful in cancer populations.^36^ Physicians should incorporate this fall risk model in clinical practice, especially in patients taking high-risk medications or patients with preexisting conditions who have a high risk of fall.

### Limitations

This study has some limitations. The degree of fall and fracture, severity of fall and fracture, clinical consequences of fall and/or fracture on therapy, and the use of bone-health agents were not reported in the primary studies. This study was unable to perform age-stratified analysis or other subgroup analyses as the primary studies were not focused on reporting risk factors for falls and fractures related to age, race, comorbidities, or geographic location. Another limitation was the lack of time-based data to calculate the fall and fracture person-year incidence rates. The majority of studies in this meta-analysis were enzalutamide-based; only 2 included apalutamide and 1 included darolutamide. Thus, it would be worthwhile to update the meta-analysis when more prospective trials are published with apalutamide and darolutamide as the results could be affected.

## Conclusions

Our study suggests that the use of ARIs is associated with a higher risk of fall and fracture. Although the incidence of fall/fracture was noted to be a higher risk in patients receiving ARIs, it is still a rare adverse event. Considering the severity of the disease and that ARIs have shown significant improvement in overall survival, the benefits may outweigh the risk of fall and fracture in some individuals. Oncologists should consider incorporating the fall-risk screening tool in older, active, patients with cancer in clinics. Appropriate use of bone-targeted agents should be considered in those patients as per established guidelines. Further prospective studies are warranted to identify potential mechanisms and to develop strategies that include a fall risk assessment tool to examine the risk factors for falls or fracture.

## Notes

Supplement. **eTable 1.** Outcomes of Reported Fall and Factures Adverse Events (AEs) in Combined Studies **eTable 2.** Clinical Heterogeneity Differences Between Studies
