# Errors in Text

**DOI:** 10.1001/jamanetworkopen.2022.55944

**Published:** 2023-01-30

In the Original Investigation titled “Evaluation of Fall and Fracture Risk Among Men With Prostate Cancer Treated With Androgen Receptor Inhibitors: A Systematic Review and Meta-analysis,”^1^ published November 17, 2020, there were errors in the text. In 4 separate places, the ARAMIS trial was misspelled. This article has been corrected.^1^
