# Supplementary material for: Evaluation of Fall and Fracture Risk Among Men With Prostate Cancer Treated With Androgen Receptor Inhibitors: A Systematic Review and Meta-analysis
Source: JAMA Netw Open. 2020 Nov 17;3(11):e2025826. doi: 10.1001/jamanetworkopen.2020.25826 (PMC7672516; doi:10.1001/jamanetworkopen.2020.25826)
Supplement: Supplement. — eTable 1. Outcomes of Reported Fall and Factures Adverse Events (AEs) in Combined Studies eTable 2. Clinical Heterogeneity Differences Between Studies [file jamanetwopen-e2025826-s001.pdf]

Supplemental Online Content

Myint ZW, Momo HD, Otto DE, Yan D, Wang P, Kolesar JM. Evaluation of fall and fracture risk among men with prostate cancer treated with androgen receptor inhibitors: a systematic review and meta-analysis. *JAMA Netw Open*. 2020;3(11):e2025826. doi:10.1001/jamanetworkopen.2020.25826

eTable 1. Outcomes of Reported Fall and Factures Adverse Events (AEs) in Combined Studies

eTable 2. Clinical Heterogeneity Differences Between Studies

This supplemental material has been provided by the authors to give readers additional information about their work.

© 2020 Myint ZW et al. *JAMA Network Open.*

eTable 1. Outcomes of Reported Fall and Factures Adverse Events (AEs) in Combined Studies

	Fall AE (ARIs arm) Total # of patients (%)		Fall AE (Control arm) Total # of patients (%)		Fracture AE (ARIs arm) Total # of patients (%)		Fracture AE (Control arm) Total # of patients (%)	
	All grades	grade ≥ 3	All grades	grade ≥ 3	All grades	grade ≥ 3	All grades	grade ≥ 3
Enzalutamide	321 (8%)	36 (0.8%)	122 (3.6%)	17 (0.5%)	75 (1.8%)	22 (0.7%)	37 (1%)	11 (0.3%)
Apalutamide	164 (12%)	18 (1.3%)	73 (8%)	7 (0.7%)	127 (10%)	29 (2.2%)	50 (5.4%)	7 (7%)
Darolutamide	40 (4.2%)	8 (0.8%)	26 (4.7)	4 (0.7%)	40 (4.2%)	9 (0.9%)	20 (3.6%)	5 (0.9%)
All studies combined	525 (8%)	62 (1%)	221 (5%)	28 (0.6%)	242 (4%)	60 (1%)	107 (2%)	23 (0.5%)

© 2020 Myint ZW et al. *JAMA Network Open.*

eTable 2. Clinical Heterogeneity Differences Between Studies

Trials	Inclusion	Exclusion	Age	Geographic location	Race	Allocation
ENZAMET	– Metastatic hormone sensitive<br>– High and low volume diseases<br>– Allowed prior docetaxel<br>Prostate adenocarcinoma	– Seizure<br>CVS (MI, CHF, arrythmia, angina) within last 3 months	– Median age 69.2 (63.2-74.5)<br>No age stratification	– Australia (57.5%)<br>– Canada (17.2%)<br>– United Kingdom (11.2%)<br>– Ireland (6.9%)<br>– New Zealand (3.6%)<br>United States (3.6%)	Missing data/not provided in the original manuscript	– Randomized<br>– Phase 3 (enzalutamide + ADT vs. placebo + ADT)<br>– Multinational
ARCHES	– Prostate adeno<br>– Metastatic<br>– Hormone sensitive<br>– Allowed <6 cycles of prior chemo	– Neuroendocrine/small cell<br>– Up to 6 cycles of chemo<br>– Major surgery within 4 weeks<br>– CVS within 3-6 months (MI, unstable angina, CHF, arrhythmias, heart block, bradycardia, hypotension, uncontrolled hypertension)	– Age <65 (25.8%)<br>– Age 65-75 (44.6%)<br>– Age ≥75 (29.6%)	– Europe (59.4%)<br>– Asia (18.1%)<br>– North America (15%)<br>– South America (5.6%)<br>– Other (1.9%)	– White (81%)<br>– Asian (13.1%)<br>– Black (1.4%)<br>Missing (4%)	– 1:1 randomized, double-blind (enzalutamide + ADT vs. placebo + ADT)<br>– Phase 3<br>– Multinational
PROSPER	– Castration resistant<br>– Non-metastatic<br>– PSA doubling time ≤10 months<br>– Use of bone targeting agent (10%)	– Seizure<br>– CVS within 3-6 months (MI< unstable angina, CHF, arrhythmias, heart block, bradycardia, hypotension, uncontrolled hypertension)	– Median age 74 (50-95)	– North America<br>– European Union<br>– Others	– Missing data/not provided in the original manuscript	– 2:1 randomization<br>– Double-blind<br>– Multinational<br>– Placebo-controlled (enzalutamide + ADT vs. placebo + ADT)<br>– Phase 3
PREVAIL	– Prostate adeno<br>– Castration resistant<br>– Metastatic<br>– No prior chemo	– Neuroendocrine/small cell<br>– Brain met<br>– Seizures<br>– CVS within 3-6 months (MI, uncontrolled angina,	– Age <65 (20%)<br>– Age 65-75 (43%)<br>– Age 75-84 (31%)	– North America (25%)<br>– Europe (52%)<br>– Others (19%)	– Asian (9.7%)<br>– Black (2.4%)<br>– White (76.7%)	– Phase 3<br>– Randomized<br>– Double-blind<br>– Multinational<br>– Placebo-controlled (enzalutamide

© 2020 Myint ZW et al. *JAMA Network Open.*

		CHF, heart block, bradycardia, hypotension, uncontrolled hypertension)	– Age ≥85 (5%)		– Other (11%)<br>–	+ ADT vs. placebo + ADT)
AFFIRM	– Prostate adeno<br>– Castration resistant<br>– Metastatic<br>– Progressed after prior docetaxel chemo<br>– Baseline bisphosphonate use (43%)<br>– Baseline ≤20 bone lesions (62%)<br>– Baseline >20 lesions (37%)	– Neuroendocrine/small cell<br>– Brain met<br>– Seizure<br>– -CVS within 3-6 months (MI, uncontrolled angina, CHF, long QT, arrhythmias, heart block, hypotension, uncontrolled hypertension, bradycardia)	– Median age 69 (41-92)<br>– Age ≥75 (25%)	– North America (33%)<br>– Others (67%)	– Missing data/not provided in the original manuscript	– Phase 3<br>– Multinational<br>– Randomized<br>– Double-blind<br>– Placebo-controlled (enzalutamide + ADT vs. placebo + ADT)
TERRAIN	– Prostate adeno<br>– Metastatic<br>– Castration resistant	– Neuroendocrine/small cell<br>– Previous chemo<br>– Brain met<br>– Seizure<br>– Previous progression on anti-androgen therapy	– Age 65 (24%)<br>– Age 65-75 (46%)<br>– Age >75 (29%)	– Europe (59%)<br>– North America (41%)	– White (92%)<br>– Black (4%)<br>– Asian (2%)	– Phase 2<br>– 1:1 randomized<br>– Double-blind (enzalutamide + ADT vs. bicalutamide + ADT)
STRIVE	– Prostate adeno<br>– Castration resistant<br>– Metastatic/non-metastatic)<br>–	– Neuroendocrine/small cell<br>– Brain met<br>– Seizures<br>– CVS risk (MI, unstable angina, CHF, arrhythmias, heart block, uncontrolled hypertension, hypotension, bradycardia)	– Age <65 (20%)<br>– Age 65-74 (41%)<br>– Age ≥75 (38%)	– Missing data/not provided in the original manuscript	– Black (15%)<br>– White (80%)<br>– Other (5%)	– Phase 2<br>– Multicenter<br>– 1:1 randomized<br>– Double blind (enzalutamide + ADT vs. bicalutamide + ADT)
PLATO	– Prostate adeno<br>– Chemo sensitive<br>– Castration resistant<br>– Metastatic<br>– Baseline bone health agent used (22-30%)<br>–	– Neuroendocrine/small cell<br>– Prior chemo<br>– Seizure<br>– -CVS risk (MI, unstable angina, CHF, arrhythmias, heart block, uncontrolled hypertension, hypotension, bradycardia)	– Age <65 (19%)<br>– Age 65-74 (40%)<br>– Age ≥75 (40%)	– North America (3%)<br>– Europe (51%)<br>– Australia (46%)	– White (87%)<br>– Black (2%)<br>– Asian (2%)	– Phase 4<br>– Randomized<br>– Double-blind<br>– Placebo controlled (enza +abiraterone vs. enza + placebo)
SPARTAN	– Prostate adeno<br>– PSA doubling time ≤10 months<br>– Non-metastatic but allow locoregional nodes <2cm	– Distant met<br>– Prior treatment with radiation, abiraterone, or anti-androgen<br>– Seizures<br>– Unstable angina<br>– Uncontrolled hypertension<br>–	– Median age 74 (48-94)	– Europe (49%)<br>– North America (35%)<br>– Asia Pacific (15.6%)	– White (65%)<br>– Asian (11.5%)<br>– Black (6%)	– Phase 3<br>– Multicenter<br>– 2:1 randomized<br>– Double blind<br>– Placebo controlled (apalutamide + ADT vs.

© 2020 Myint ZW et al. *JAMA Network Open.*

	– Castration resistant<br>– Baseline used of bone health agents (10%)					placebo + ADT)
TITAN	– Prostate adeno<br>– Metastatic<br>– Hormone sensitive<br>– Allowed prior chemo	– Neuroendocrine/small cell<br>– Brain met<br>– Seizure<br>– Uncontrolled hypertension<br>– Unstable angina/MI,CHF, thromboembolic, arrhythmias	– Age <65 (28%)<br>– Age 65-74 (46%)<br>– Age ≥75 (25%)	– North America & European Union (33%)<br>– Other (67%)	– White (67%)<br>– Asian (22.7%)<br>– Black (1.9%)<br>– Others (6%)	– Phase 3<br>– Multicenter<br>– Randomized<br>– Placebo controlled (Apalutamide + ADT vs. placebo + ADT)
ARMIS	– Prostate adeno<br>– Castration resistant<br>– PSA doubling time ≤10 months<br>– Non-metastatic but allowed loco-regional node <2cm<br>– Baseline used of bone targeting agent (7%)	– Neuroendocrine/small cell<br>– Distant met<br>– Stroke, MI, unstable angina, CAD, PAD, CABG, CHF<br>– Uncontrolled hypertension<br>–	– Age <65 (20%)<br>– Age 65-74 (62%)<br>– Age 75-84 (62%)<br>– Age ≥85 (14%)	– North America (11%)<br>– Asia Pacific (12%)<br>– Others (76%)	– Missing data/not provided in the original manuscript	– Phase 3<br>– Randomized<br>– Double blind<br>– Placebo controlled (Darolutamide + ADT vs. placebo + ADT)

Acronyms: CVS = cardiovascular disease, MI = myocardial infarction, CHF = congestive heart failure, ADT = androgen deprivation therapy, PSA = prostatic specific antigen, PAD = peripheral artery disease, CABG = coronary artery bypass graft

© 2020 Myint ZW et al. *JAMA Network Open.*
